# Increased Expression of Myeloid-Derived Suppressor Cells in Patients with HBV-Related Hepatocellular Carcinoma

**DOI:** 10.1155/2020/6527192

**Published:** 2020-03-14

**Authors:** Tianyu Li, Xinyu Zhang, Zhuo Lv, Li Gao, Huimin Yan

**Affiliations:** ^1^Graduate College of Hebei Medical University, Hebei Medical University, Shijiazhuang, Hebei 050017, China; ^2^College of Life Sciences, Hebei Normal University, Shijiazhuang, Hebei 050024, China; ^3^Clinical Research Center, Shijiazhuang Fifth Hospital, Shijiazhuang, Hebei 050021, China

## Abstract

**Methods:**

The percentages of MDSCs, IFN-*γ*-producing CD4 and CD8 T cells in the peripheral blood of HCC patients, chronic hepatitis B (CHB) patients, and healthy controls (HC) were determined by flow cytometry. The serum concentrations of IL-10 and TNF-*α* were determined by ELISA. The association of the percentages of MDSCs with tumor burden, liver function parameters, systemic inflammation-related indexes, and IFN-*γ*-producing T cells was assessed.

**Results:**

The percentages of MDSCs and PMN-MDSCs were significantly higher in HCC patients than those in CHB patients and HC. The level of MDSCs was correlated with indirect bilirubin and prealbumin, as well as systemic inflammation response index, monocyte/lymphocyte ratio, and monocyte counts. The frequency of IFN-*γ*-producing CD8 T cells of HCC patients was lower than that of HC. However, there was no relationship between MDSCs and IFN-*γ*-producing CD8 T cells. The level of IL-10 in HCC patients was significantly higher than that in CHB patients.

**Conclusion:**

MDSCs seem to play an important role in the process leading from chronic HBV infection to HCC. Early inhibiting these cells could affect tumor progression.

## 1. Introduction

Hepatocellular carcinoma (HCC) is the most common primary liver cancer and the most important cause of death in patients with cirrhosis [1]. Epidemiological studies indicate that chronic infection with hepatitis B virus (HBV) is the leading cause of HCC in developing countries, and the incidence of HCC caused by chronic hepatitis B (CHB) is over 40% in East Asia [2]. Accumulating evidence has shown that suppression of tumor immune surveillance is one of the important mechanisms for the occurrence and progression of HCC. In the processes of tumor development, hepatoma cells can drive the recruitment and expansion of immunosuppressive cells, which result in suppression of antitumor immunity [3, 4].

Myeloid-derived suppressor cells (MDSCs) are important immunosuppressive cells derived from myeloid progenitor cells. These cells are expanded under pathological conditions and have been implicated as key components of the tumor microenvironment [5, 6]. MDSCs can facilitate tumor progression and metastasis through inhibiting antitumor immune effector cells [7, 8] and nonimmunological mechanisms [9]. Recently, a few studies have reported the involvement of aberrant expression of MDSCs in the pathological process of HCC patients [10–12]. However, due to the difference of cell sample types and phenotypic heterogeneity of human MDSCs, the results of these studies were inconsistent and controversial.

In the present study, we analyzed the frequency of MDSCs in peripheral blood of HBV-related HCC patients using HLA-DR^-/low^CD11b^+^CD33^+^ as the marker for MDSCs. We also evaluated the relationship between MDSCs and clinicopathological parameters, as well as Th1 and Tc1 cells.

## 2. Materials and Methods

### 2.1. Patients

A total of 48 newly diagnosed HBV-related HCC patients, 16 CHB patients, and 21 healthy controls (HC) were recruited at the Shijiazhuang Fifth Hospital, China, from April 2017 to January 2019. The diagnosis of HCC was based on pathology or clinical presentation with typical imaging findings (computed tomography and/or magnetic resonance imaging). Of these patients, 26 (54.2%) received antiviral therapy, which included the medication of entecavir, tenofovir, lamivudine, and adefovir dipivoxil. The diagnosis of CHB was based on clinical and laboratory findings according to the Guideline of Prevention and Treatment for Chronic Hepatitis B (2015 version) issued by the Chinese Society of Hepatology. HC individuals had normal parameters of liver function test and blood routine test. The characteristics of all subjects enrolled in this study are listed in [Supplementary-material supplementary-material-1]. There was no significant difference between CHB and HCC patients. Blood samples were collected before the treatments. The study has been approved by the Shijiazhuang Fifth Hospital Ethics Committee.

### 2.2. Isolation of Peripheral Blood Mononuclear Cells (PBMCs)

EDTA-anticoagulated venous blood samples were collected from study subjects, and the experimental treatment was carried out within 4 hours. Peripheral blood mononuclear cells (PBMCs) were separated using a density gradient centrifugation by human lymphocyte separation medium (Solarbio Science & Technology, Beijing, China).

### 2.3. Flow Cytometric Analysis

To investigate the percentages of MDSCs, 200 *μ*L of whole blood sample was stained with monoclonal antibodies, including HLA-DR-PE/Cy7, CD11b-APC, CD33-PE, CD14-FITC, and CD15-PerCp5.5 (BioLegend, San Diego, CA, USA). The cells were incubated at room temperature for 15 min and then were treated with 1 mL of lysing solution (Beckman Coulter, Miami, FL, USA) at room temperature for 10 min. Finally, samples were washed twice and analyzed with flow cytometry.

To investigate the percentages of IFN-*γ*-producing T cells, PBMCs (1 × 10^6^) were cultured with phorbol-12-myristate 13-acetate (50 ng/mL, Multi Sciences, Hangzhou, Zhejiang, China), ionomycin (1 *μ*g/mL, Multi Sciences), and GolgiStop (Becton Dickinson, San Diego, CA, USA) at 37°C for 4 h. The cells were stained with CD4-FITC and CD8a-PerCP/Cy5.5 (BioLegend) and then fixed and permeabilized with Fixation and Permeabilization Solution (Becton Dickinson). After staining with IFN-*γ*-PE/Cy7 (BioLegend), all samples were analyzed using FACSCanto^II^ flow cytometer with FACSDiva software (BD Biosciences, San Jose, CA).

### 2.4. Biochemical Assessments

Liver function and blood routine test were measured using automated analyzers with standard techniques. Four inflammation-based scores were defined as follows: MLR = monocyte/lymphocyte, NLR = neutrophil/lymphocyte, PLR = platelet/lymphocyte, and systemic inflammation response index (SIRI) = neutrophil×monocyte/lymphocyte. Serum HBV DNA load was determined by real-time PCR, and the lowest detection limit was 500 copies/mL. Serum HBeAg and AFP were determined by electrochemiluminescence immunoassay.

### 2.5. Enzyme-Linked Immunosorbent Assay (ELISA) Analysis

Serum samples were collected and kept at −80°C. The concentration of IL-10 and TNF-*α* was measured by ELISA using OptEIA kits (BD Bioscience, USA) according to the manufacturer's instructions. All samples were assayed in duplicate.

### 2.6. Statistical Analysis

All statistical analyses were carried out by SPSS 21.0 software (SPSS Inc., Chicago, IL, USA). Data were expressed as mean ± SD or median/IQR. The significance of differences was tested using the Kruskal-Wallis H test. Pearson correlation and Spearman correlation tests were done for correlation analysis. Subsequently, multiple linear regression analysis was performed on factors with statistical significance. A two-sided *P* < 0.05 was considered to be statistically significant.

## 3. Results

### 3.1. The Frequency of Circulating MDSCs and Their Subsets in HCC Patients

**Figure 1:**
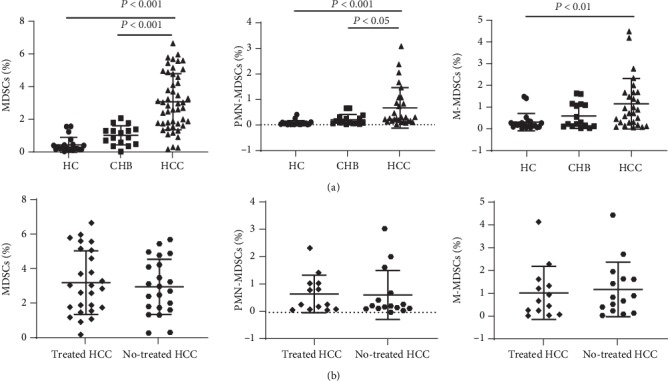
Frequency of circulating MDSCs in study subjects. (a) Frequency of MDSCs, PMN-MDSCs, and M-MDSCs in HCC patients, CHB patients, and healthy controls. (b) Frequency of MDSCs, PMN-MDSCs, and M-MDSCs in HCC patients who received and did not receive anti-HBV therapy.

As shown in Figure 1(a), the frequencies of both MDSCs and PMN-MDSCs in HCC patients were significantly increased compared with CHB patients and HC. The frequency of M-MDSCs in HCC patients was significantly increased compared with HC, but there was no significant difference between HCC patients and CHB patients. To investigate whether the change in MDSC level was due to the effect of anti-HBV therapy before HCC occurrence, HCC patients were divided into two groups according to whether they received antiviral therapy, and the percentages of MDSCs and their subsets were analyzed. The results showed that there was no significant difference between patients who received and did not receive anti-HBV therapy (Figure 1(b)).

### 3.2. The Association between Circulating MDSCs and Clinicopathologic Characteristics in HCC Patients

HCC patients were divided into two groups according to clinicopathologic characteristics, and the frequencies of MDSCs were compared. The results showed that there were no significant differences in the proportion of MDSCs between patients with different disease states (Table 1). We further evaluated the prognostic value of MDSCs, but no association was observed between MDSC level and overall survival ([Supplementary-material supplementary-material-1]).

### 3.3. The Association between Circulating MDSCs and Liver Damage in HCC Patients

**Figure 2:**
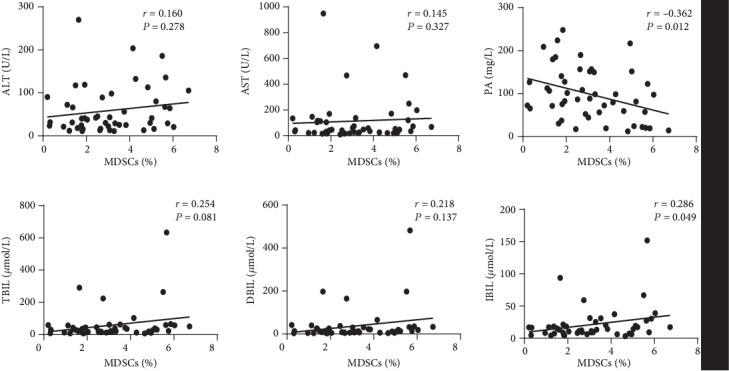
Correlation between the percentages of circulating MDSCs and levels of liver function parameters. Liver function parameters including alanine aminotransferase (ALT), aspartate aminotransferase (AST), prealbumin (PA), total bilirubin (TBIL), direct bilirubin (DBIL), and indirect bilirubin (IBIL).

The frequencies of MDSCs were positively correlated with the levels of indirect bilirubin (IBIL) and negatively correlated with the levels of prealbumin (PA). However, no correlation was found between the frequencies of MDSCs and other liver function parameters (Figure 2). Moreover, the frequencies of PMN-MDSCs were only positively correlated with levels of IBIL. No significant correlation between M-MDSCs and any of liver function parameters ([Supplementary-material supplementary-material-1]).

### 3.4. The Association between Circulating MDSCs and Systemic Inflammation-Related Indexes in HCC Patients

**Figure 3:**
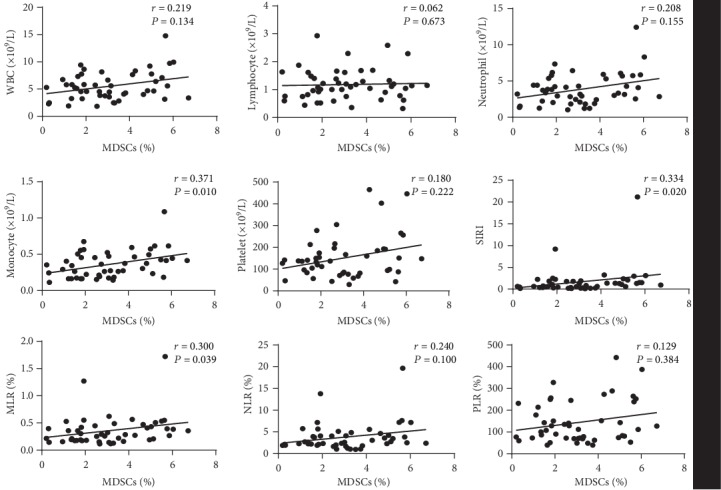
Correlations between the percentages of circulating MDSCs and levels of system inflammation parameters. Levels of system inflammation parameters including the counts of WBC, lymphocyte, neutrophil, monocyte, and platelet, as well as systemic inflammation response index (SIRI), monocyte/lymphocyte (MLR), neutrophil/lymphocyte (NLR), and platelet/lymphocyte (PLR).

Peripheral blood leukocyte counts and ratios were measured, and the relationship between MDSCs and these indexes was assessed. The results showed that the percentages of MDSCs were positively correlated with SIRI, MLR, and the counts of monocyte (Figure 3). There was no correlation between MDSCs and the counts of leukocyte, lymphocyte, neutrophil, platelet, NLR, and PLR. Moreover, neither PMN-MDSCs nor M-MDSCs showed a correlation with any of systemic inflammation-related indexes ([Supplementary-material supplementary-material-1]).

We further performed a multiple linear regression analysis to investigate the relationship between MDSC level and different factors that had significant correlation as independent variable. The results showed that PA and monocytes were significantly associated with MDSC level (Table 2).

### 3.5. The Association between MDSCs and IFN-*γ*-Producing CD4 and CD8 T Cells in HCC Patients

**Figure 4:**
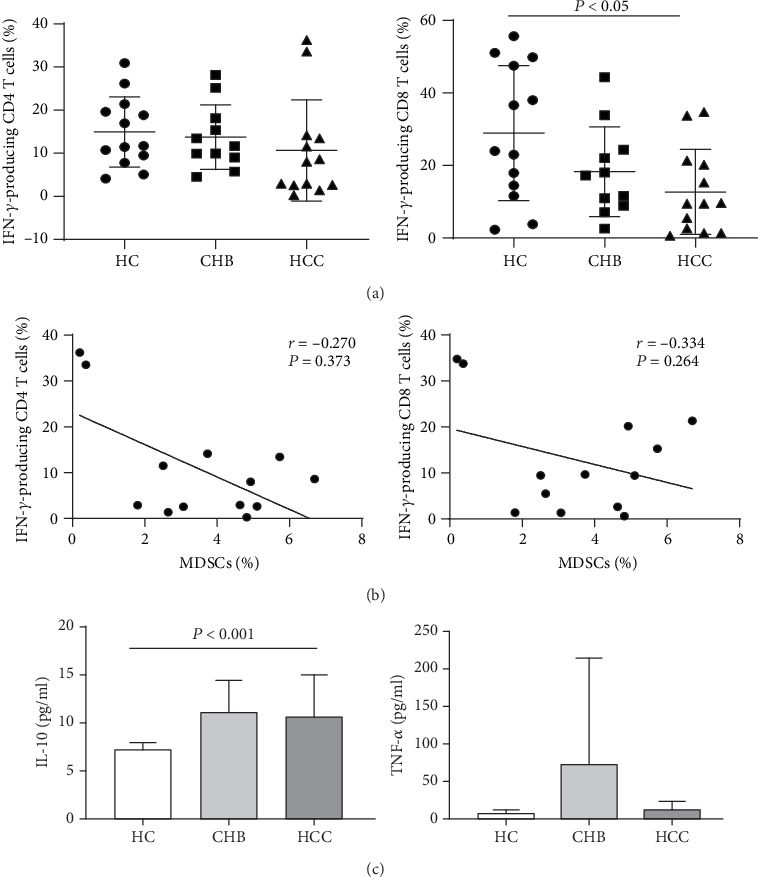
Frequency of IFN-*γ*-producing CD4 and CD8 T cells and their relationships with MDSCs. (a) The percentages of IFN-*γ*-producing CD4 and CD8 T cells in HCC patients, CHB patients, and healthy controls. (b) Correlation between MDSCs and IFN-*γ*-producing CD4 and CD8 T cells. (c) The serum level of IL-10 and TNF-*α* in HCC patients, CHB patients, and healthy controls.

The percentage of IFN-*γ*-producing CD4 and CD8 cells was determined by FACS, and representative dot plots were shown in [Supplementary-material supplementary-material-1]. The percentages of IFN-*γ*-producing CD8 T cells of HCC patients were significantly lower than those of HC. However, there was no significant difference in the percentages of IFN-*γ*-producing CD4 T cells among the three groups (Figure 4(a)). Considering the immunosuppressive role of MDSCs on IFN-*γ*-producing T cells, the relationship was analyzed. The results showed that there were no statistical correlations between the percentages of MDSCs and IFN-*γ*-producing CD4 or CD8 T cells (Figure 4(b)). We further observed the level of IL-10 and TNF-*α*. The results showed that the level of IL-10 in HCC patients was significantly higher than that in HC. However, there was no significant difference in the level of TNF-*α* among the three groups (Figure 4(c)).

## 4. Discussion

Dysregulated immune response is considered an important mechanism for the occurrence and progression of tumor. As one of the important components of the immunosuppressive network, MDSCs have been shown to facilitate tumor formation by blocking the host immune system. A few studies have investigated the involvement of MDSCs in HCC patients. However, there are several controversial issues requiring attention. First, different markers were used to identify MDSCs. Some studies have described MDSCs as HLA-DR^-/low^CD14^+^ [7, 10, 13], whereas some studies have defined MDSCs based on the expression of CD11 and CD33 [12, 14]. Recently, HLA-DR^-/low^CD14^+^ cells are considered as monocytic MDSCs [15, 16]; therefore, we examined the frequencies of HLA-DR^-/low^CD11b^+^CD33^+^ in this study. Second, the selection of study population is inconsistent. HCC patients involved in previous studies included those associated with chronic infection with HBV, HCV, alcoholic liver disease, and nonalcoholic steatohepatitis. The level of MDSCs might be varied in different subtypes of HCC because there are different immune mechanisms underlying the pathogenesis of disease. The vast majority of patients with HCC have an available case history of chronic HBV infection in China [17]. Therefore, this study focused on the HBV-related HCC. Third, there existed the heterogeneity of sample sources. Most of the studies analyzed MDSCs in the PBMC, which may lead to a loss of high-density polymorphonuclear neutrophil during the process of density gradient separation [18]. In the present study, we used whole blood to detect the frequency of circulating MDSCs in a cohort of patients with HBV-related HCC. Our data demonstrated that the frequency of MDSCs in HCC patients was significantly higher than that in CHB patients and HC. We also found that there was an increased percentage of MDSC in CHB patients compared with HC, indicating that no matter tumor microenvironment or inflammatory environment induces the expansion of MDSCs.

We further analyzed the relationship between MDSCs and clinicopathological features. The results revealed that MDSCs did not associate with any parameters of tumor burden and overall survival but positively correlated with several markers for hepatic injury. Evidence of our current study and previous data have shown the involvement of MDSCs in the pathogenesis of chronic HBV infection [19, 20]. Taken together, compared to the role of promoting HCC progression, MDSCs seem to play a more important role in the process leading from infection to cancer. These cells induced by chronic HBV infection might contribute to the establishment of protumorigenic microenvironment and thereby facilitate malignant progression of HCC. In China, chronic HBV infection is the leading cause of HCC. Targeted therapy for MDSCs in the stage of HBV infection may be one of the effective methods to prevent HBV-related HCC.

There is growing evidence that chronic inflammation is closely related to cancer. Host inflammatory responses can promote carcinogenesis and tumor progression. Several hematologic inflammation markers, such as SIRI, MLR, NLR, PLR, neutrophil, and monocyte counts, have been recognized as prognostic factors in various cancers, including HCC [21, 22]. MDSCs have also been described to be a prognostic marker for HCC patients [13]. However, few studies have assessed the relationship between MDSCs and these inflammation markers. In the present study, we found that the frequencies of MDSCs positively corrected with monocyte counts, SIRI, and MLR, two scores closely related to monocytes. Multivariate analysis showed that monocytes significantly related with MDSCs. A similar result has been found in a previous report [23]. These data suggest that MDSC level was associated with systemic inflammation in HCC patients. Emerging evidence has shown that the monocyte population in cancer patients consists of conventional monocytes and monocytic MDSCs [6, 24]. It is likely that chronic HBV infection triggers the generation and expansion of these two populations, and thereby the crosstalk between each other leads to a further increase. Although MDSCs have been reported to associate with protective functions in the context of acute liver inflammation, these cells seem to facilitate inflammation and tissue damage during chronic liver diseases. It was reported that HBV induces the expansion and activation of MDSCs [25], which favors T cell exhaustion. This leads to the establishment of persistent HBV infection and the maintenance of chronic inflammation. The interplay between HBV and host inflammatory factors contributes to the development of HCC.

IFN-*γ*-producing T cells are important antitumor immune cells, which control tumor growth by producing IFN-*γ* [26]. However, during tumor progression, tumor cells can escape from immune surveillance by limiting antitumor immunity. In established HCC, decreased secretion of IFN-*γ* by T cells has been reported [27]. In agreement with the previous studies, the result of this study revealed that both IFN-*γ*-producing CD4 and CD8 T cells were reduced in HCC patients, suggesting that T cells were impaired during the development of HCC. We further assessed the association between MDSCs and IFN-*γ*-producing T cells. Although it did not reach statistical significance, there was a tendency toward negative correlation. MDSCs can suppress T cell proliferation and activation and promote T cell apoptosis by various mechanisms [28–31]. We speculate that the decrease of IFN-*γ*-producing cells may be in part due to the participation of MDSCs. Other multiple inhibitory factors, such as regulatory T cells, inhibitory molecules expressed on T cells, and inhibitory cytokines, may also play an important role. In this study, we have not attempted to evaluate the ability of MDSCs to suppress T cell immune responses. Therefore, it is different to conclude that whether the decrease of IFN-*γ*-producing cells is due to the direct suppressive function of MDSCs. Further research is required to address this limitation.

In summary, the current study revealed that the frequency of circulating MDSCs was significantly increased. Furthermore, MDSCs positively correlated with liver damage and systemic inflammation in HBV-related HCC patients. Our data suggest that the increased MDSCs may accelerate the disease progression from chronic hepatitis to HCC. Therefore, the reduction or elimination of MDSCs in CHB patients may prevent or delay the development of HCC.

## Figures and Tables

**Table 1:** Relationships between the frequency of MDSCs and clinical characteristics of HCC patients.

Characteristics	*N*	MDSCs (%)	*P*
Gender			
Male	37	3.22 ± 1.77	0.439
Female	11	2.75 ± 1.59	
Age			
≤50	19	3.39 ± 1.84	0.337
>50	29	2.93 ± 1.65	
Tumor size			
≤5 cm	22	3.09 ± 1.75	0.934
>5 cm	26	3.13 ± 1.74	
Tumor number			
Single	13	2.99 ± 1.58	0.776
Multiple	35	3.16 ± 1.80	
Tumor metastasis			
No	22	3.06 ± 1.38	0.852
Yes	26	3.15 ± 2.00	
BCLC stage			
0/A/B	22	3.06 ± 1.38	0.852
C/D	26	3.15 ± 2.00	
Liver cirrhosis			
Compensation	14	2.70 ± 1.40	0.295
Decompensation	34	3.28 ± 1.84	
Child-Pugh			
A	16	2.78 ± 1.42	0.353
B/C	32	3.28 ± 1.86	
Ascites			
No	11	2.99 ± 1.43	0.786
Yes	37	3.15 ± 1.82	
HBeAg			
+	34	3.28 ± 1.70	0.298
-	14	2.70 ± 1.78	
HBV-DNA			
≤2000 copies/mL	35	3.24 ± 1.72	0.410
>2000 copies/mL	13	2.77 ± 1.77	
AFP			
≤400 ng/mL	26	3.37 ± 1.73	0.260
>400 ng/mL	22	2.81 ± 1.71	

**Table 2:** Multivariate analysis between the frequencies of MDSCs and clinicopathological parameters.

Parameters	*β*	*P* value
PA	-0.335	0.013
IBIL	0.155	0.303
Monocytes	1.257	0.007
SIRI	-0.084	0.693
MLR	-0.043	0.832

## Data Availability

The data used to support the findings of this study are available from the corresponding author upon request.

## Supplementary Materials

Supplementary Materials Figure S1: the association between MDSC level and overall survival. Figure S2: gating strategy of IFN-*γ*-producing CD4 and CD8 T cells by flow cytometry analysis. Table S1: characteristics of study subjects. Table S2: correlation between the frequencies of both PMN-MDSCs and M-MDSCs and levels of liver function parameters. Table S3: correlation between the frequencies of both PMN-MDSCs and M-MDSCs and levels of system inflammation parameters.
