# Supplementary material for: Increased Expression of Myeloid-Derived Suppressor Cells in Patients with HBV-Related Hepatocellular Carcinoma
Source: Biomed Res Int. 2020 Mar 14;2020:6527192. doi: 10.1155/2020/6527192 (PMC7097855; doi:10.1155/2020/6527192)
Supplement: Supplementary Materials — Figure S1: the association between MDSC level and overall survival. Figure S2: gating strategy of IFN-γ-producing CD4 and CD8 T cells by flow cytometry analysis. Table S1: characteristics of study subjects. Table S2: correlation between the frequencies of both PMN-MDSCs and M-MDSCs and levels of liver function parameters. Table S3: correlation between the frequencies of both PMN-MDSCs and M-MDSCs and levels of system inflammation parameters. [file 6527192.f1.doc]

Figure S1 The association between MDSCs level and overall survival.


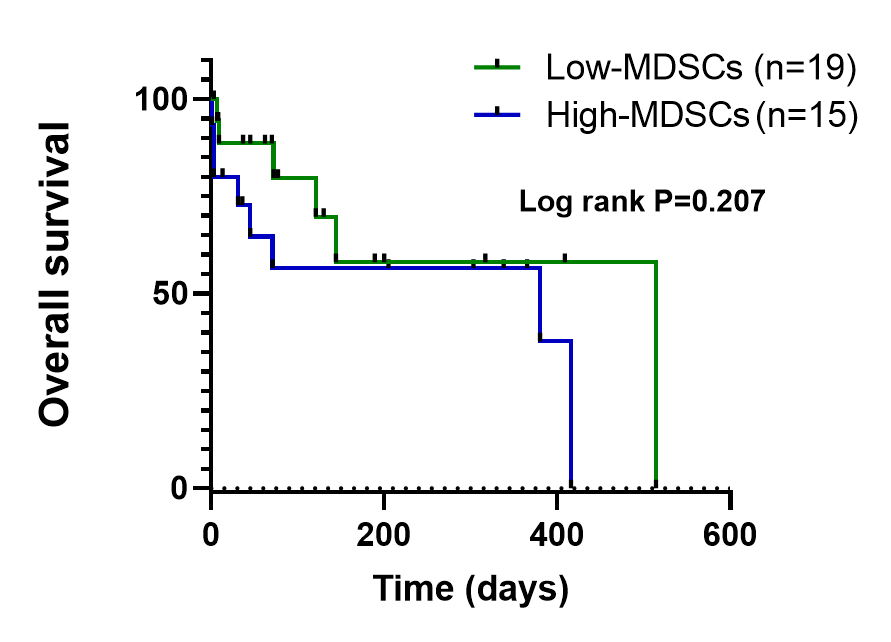


Figure S2 Gating strategy of IFN-γ-producing CD4 and CD8 T cells by flow cytometry analysis.
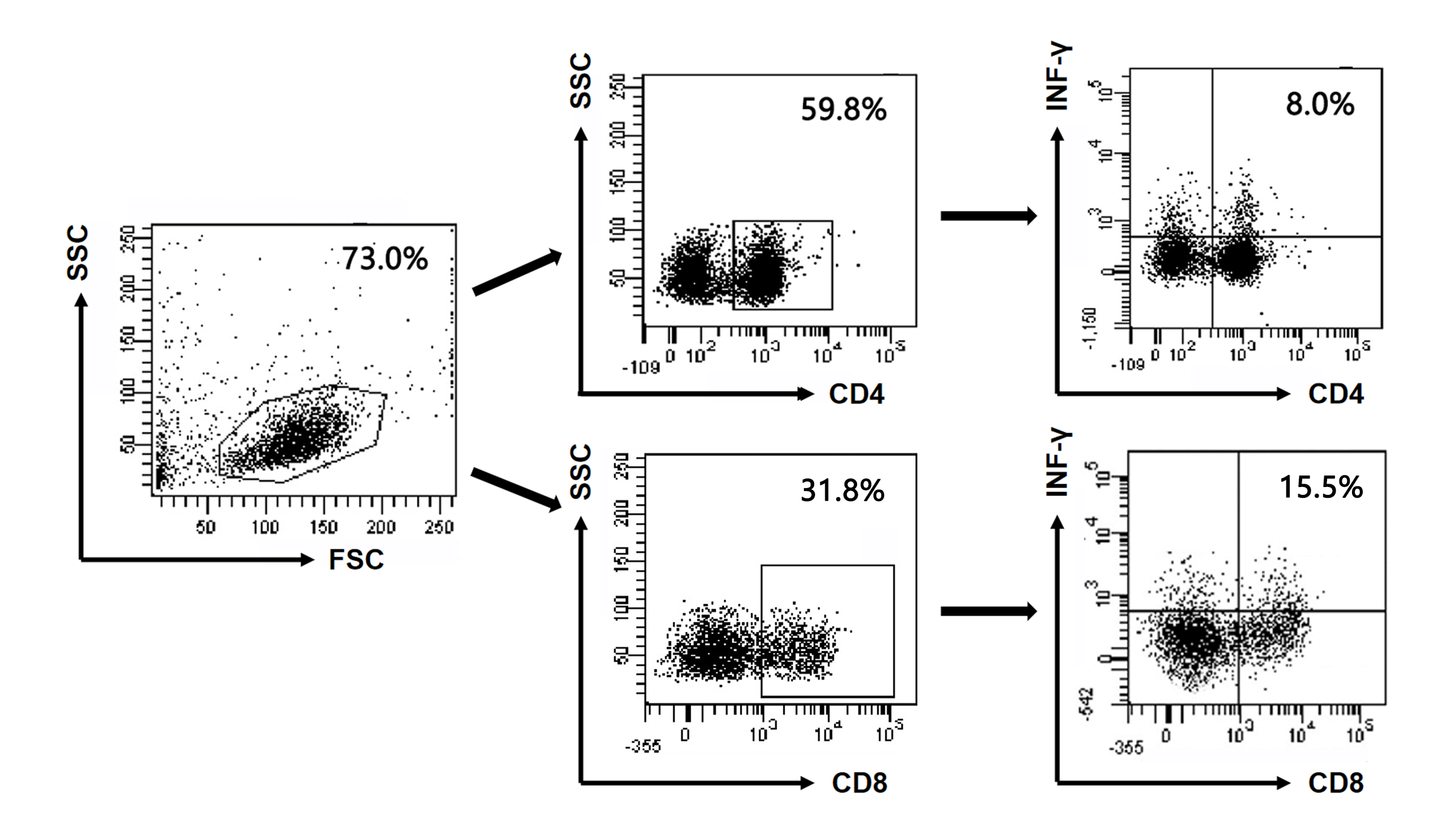


Table S1 Characteristics of study subjects

| Group | HC | CHB | HCC |
| --- | --- | --- | --- |
| Male/Female | 14/7 | 11/5 | 37/11 |
| Age (years) | 50.4± 6.1 | 47.0 ± 11.1 | 49.2 ± 7.5 |
| ALT (U/L) | 15.8 (10.6, 29.4) | 52.5 (43.3, 148.0) | 42.5 (30.0,77.8) |
| AST (U/L) | 19.6 (16.3, 23.8) | 45.0 (32.0, 78.8) | 49.8 (26.9, 122.0) |
| TBIL (μmol/L) | 11.2 (9.9, 14.7) | 20.0(13.5, 33.0) | 29.9 (15.6, 55.0) |
| DBIL (μmol/L) | 3.8 (3.2, 4.9) | 10.0 (5.3, 13.0) | 13.0 (6.3,31.1) |
| IBIL (μmol/L) | 7.2 (6.2, 9.9) | 11.0 (6.5, 21.0) | 14.0 (8.3, 20.8) |
| PTA (%) | ND | 79.0 ± 14.3 | 71.8 ± 17.7 |
| INR | ND | 1.03 ± 0.12 | 1.2 (1.05, 1.30) |
| APTT (s) | ND | 37.2 ± 6.4 | 33.7 (29.0, 40.2) |
| HBeAg (+/-) | 0/0 | 12/4 | 34/14 |

HC, healthy control; CHB, chronic hepatitis B; ALT, alanine aminotransferase; AST, aspartate aminotransferase; TBIL, total bilirubin; DBIL, direct bilirubin; IBIL, indirect bilirubin; PTA, prothrombi time activity; INR, international normalized ratio; APTT, activated partial thromboplastin time; ND, not determined.

Table S2 Correlation between the frequencies of both PMN-MDSCs and M-MDSCs and levels of liver function parameters.

| Group | PMN-MDSCs (%) | | M-MDSCs (%) | |
| --- | --- | --- | --- | --- |
|  | r | P | r | P |
| ALT (U/L) | 0.076 | 0.702 | 0.249 | 0.202 |
| AST (U/L) | 0.273 | 0.158 | 0.021 | 0.916 |
| PA (mg/L) | -0.361 | 0.059 | 0.153 | 0.438 |
| TBIL (μmol/L) | 0.354 | 0.065 | 0.123 | 0.533 |
| DBIL (μmol/L) | 0.294 | 0.129 | 0.182 | 0.355 |
| IBIL (μmol/L) | 0.393 | 0.039^*^ | 0.193 | 0.32 |

Table S3 Correlation between the frequencies of both PMN-MDSCs and M-MDSCs and levels of system inflammation parameters.

| Group | PMN-MDSCs (%) | | M-MDSCs (%) | |
| --- | --- | --- | --- | --- |
|  | r | P | r | P |
| WBC (×10^9^/L) | 0.237 | 0.226 | 0.141 | 0.474 |
| Neutrophil (×10^9^/L) | 0.315 | 0.102 | 0.159 | 0.418 |
| Lymphocyte (×10^9^/L) | -0.045 | 0.820 | -0.136 | 0.492 |
| Monocyte (×10^9^/L) | 0.181 | 0.356 | 0.186 | 0.344 |
| Platelet (×10^9^/L) | 0.056 | 0.776 | 0.051 | 0.797 |
| MLR (%) | 0.281 | 0.148 | 0.251 | 0.197 |
| NLR (%) | 0.306 | 0.052 | 0.230 | 0.239 |
| PLR (%) | 0.039 | 0.842 | 0.146 | 0.458 |
| SIRI | 0.340 | 0.077 | 0.235 | 0.228 |
